# Succinylation in cancer immunotherapy: mechanisms, biomarkers, and therapeutic implications

**DOI:** 10.3389/fimmu.2026.1794817

**Published:** 2026-05-08

**Authors:** Pan Xie, Ming-Hui Long, Zhao-Qian Liu, Jing Wang, De-Hua Liao, Zhi-Bin Wang

**Affiliations:** 1 Department of Pharmacy, The Affiliated Cancer Hospital of Xiangya School of Medicine, Central South University/ Hunan Cancer Hospital, Changsha, China; 2 Department of Clinical Pharmacology, Hunan Key Laboratory of Pharmacogenetics, and National Clinical Research Center for Geriatric Disorders, Xiangya Hospital, Central South University, Changsha, Hunan, China; 3 Institute of Clinical Pharmacology, Engineering Research Center for applied Technology of Pharmacogenomics of Ministry of Education, Central South University, Changsha, Hunan, China; 4 Hunan Gynecological Tumor Clinical Research Center; Hunan Key Laboratory of Cancer Metabolism; Hunan Cancer Hospital, and the Affiliated Cancer Hospital of Xiangya School of Medicine, Central South University, Changsha, China

**Keywords:** cancer immunotherapy, metabolic reprogramming, succinylation, targeted therapy, tumor immune microenvironment

## Abstract

Succinylation, a dynamic post-translational modification characterized by the addition of a succinyl moiety to lysine residues, has emerged as a pivotal regulator at the interface of metabolic reprogramming and immune surveillance in cancer. This review systematically delineates the molecular mechanisms and therapeutic implications of succinylation in cancer immunotherapy. We detail the writers (e.g., CPT1A, KAT2A), erasers (e.g., SIRT5, SIRT7), and readers that constitute the enzymatic systems governing its dynamics, as well as its profound impact on core metabolic networks including the TCA cycle and glycolysis, thereby fueling tumor progression. Crucially, succinylation has been shown to regulate the tumor immune microenvironment by regulating immune checkpoint stability (e.g., promoting PD-L1 degradation), shaping the polarization and function of macrophages, dendritic cells, and T cells, and influencing immunogenic cell death. These modifications create a complex duality, capable of both enhancing anti-tumor immunity and facilitating immune evasion. We further summarize emerging therapeutic strategies, including small-molecule inhibitors targeting succinylation enzymes, metabolic interventions, and combination therapies designed to harness this pathway to overcome immunotherapy resistance. Finally, we discuss current challenges such as the incomplete mapping of enzyme-substrate relationships and the spatiotemporal heterogeneity of modifications within tumors, while highlighting future directions integrating CRISPR screening, AI prediction models, and single-cell multi-omics to advance precision targeting of succinylation for innovative cancer immunotherapies.

## 1 Introduction

Post-translational modifications (PTMs) refer to the alteration of protein biochemical properties through the covalent attachment or removal of chemical groups on one or more amino acid residues, under the regulation primarily driven by enzymatic actions (1). This process encompasses various types, including phosphorylation, glycosylation, ubiquitination, nitrosylation, methylation, acetylation, and lipidation, and impacts nearly all aspects of cellular biology and pathology (such as signal transduction, cell structure, metabolism, and immune responses) (2, 3). In signal transduction, PTMs regulate the activity of signaling pathways via their molecular switch properties. For instance, phosphorylation modification-the p38 MAPK cascade (4) -enables rapid signal amplification through kinase-mediated activation loops; in contrast, ubiquitination (e.g., the endocytosis and degradation of EGFR (5)) terminates signal transduction via the proteasomal pathway. The reversibility of these modifications ensures the spatiotemporal precision of intracellular signal responses. In metabolic reprogramming, PTMs can also regulate nutrient status to meet biosynthetic demands: for example, acetylation of ACLY balances glycolysis and lipid synthesis by modulating enzyme activity, while O-GlcNAc modification coordinates glucose metabolism and transcriptional programs through competing with phosphorylation sites (6). These modifications allow cells to dynamically restructure metabolic networks under stress conditions such as hypoxia or oncogenic signaling. Of particular importance, PTMs orchestrate the regulation of immune checkpoints: processes including ubiquitination and ubiquitin-like modifications can negatively regulate PD-1/PD-L1 activity, whereas deubiquitination, glycosylation, and palmitoylation exert positive regulatory effects (7). Dysregulation of these modifications is closely associated with tumor immune evasion, making PTM-targeted therapies a critical strategy in cancer immunotherapy (8, 9).

**Figure 1:**
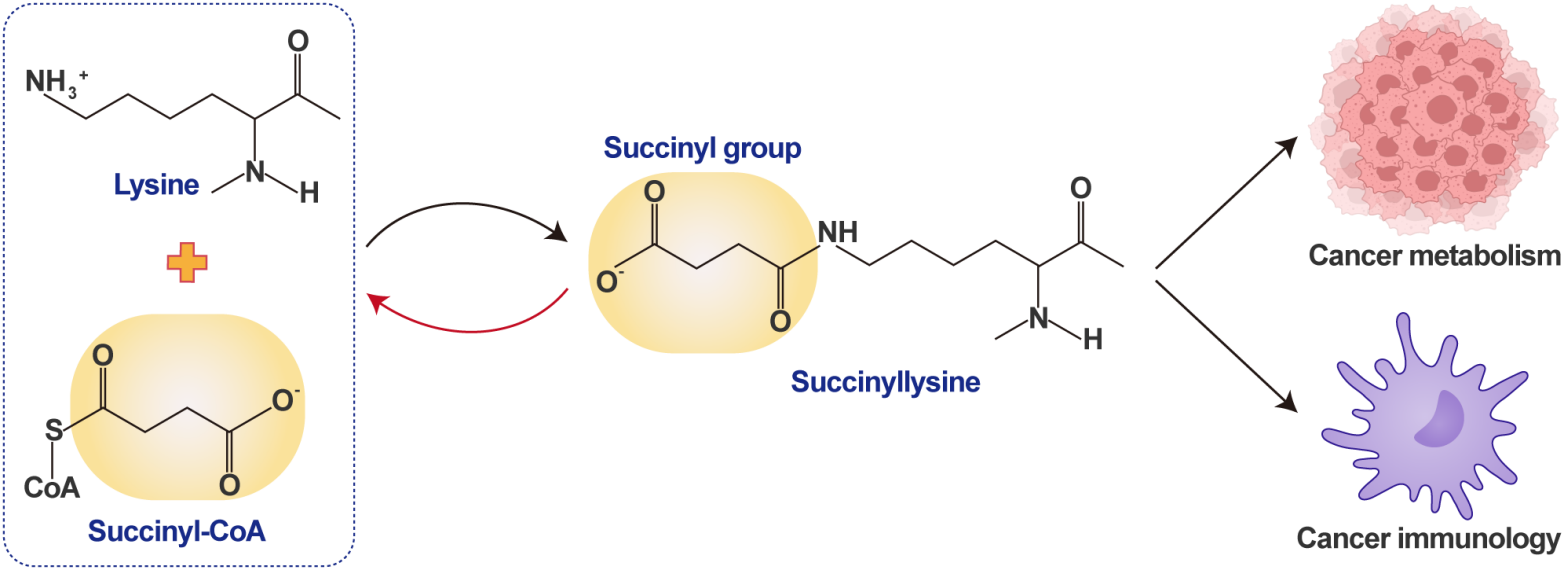
Overview of succinylation in cancer immunotherapy: bridging metabolism and immunity. A lysine residue covalently binds a succinyl group from succinyl-CoA to form succinyllysine. As a critical post-translational modification, succinylation acts as a core bridge linking cancer metabolism and immunity, modulating protein functions to regulate cancer-associated metabolic and immune processes. Succinyl-CoA, Succinyl coenzyme A; CoA, Coenzyme A.

Succinylation, a newly identified type of post-translational modification (PTM) discovered in recent years, refers to a biochemical process in which a succinyl group (i.e., a butanedioic acid group) is covalently attached to specific amino acid residues of a protein-typically lysine residues-thereby significantly altering the protein’s charge distribution and spatial conformation (**Figure 1**).

Since its first identification in histones in 2010, succinylation has rapidly emerged as a research hotspot in metabolism and disease regulation, largely due to its unique chemical properties: the introduction of negative charges and large molecular groups (10). Studies on succinylomics (the study of succinylation modifications) rely on highly efficient specific enrichment techniques and high-throughput detection technologies. Currently, quantitative strategies combining immunoprecipitation with succinylation-specific antibodies and liquid chromatography-tandem mass spectrometry (LC-MS/MS) have become the gold-standard method for identifying succinylation sites (11). However, traditional omics techniques have struggled to effectively resolve the cellular heterogeneity of succinylation modifications. In recent years, the integration of techniques such as subcellular localization analysis and single-cell multi-omics has provided a new perspective for exploring the spatial distribution and regulatory mechanisms of succinylation (12). This multimodal analytical strategy not only enables the localization of modified hotspots but also correlates the metabolic status and phenotype of specific cell subsets, offering crucial insights for understanding the role of succinylation in tumor microenvironment remodeling (13, 14).

Succinylation is widely present in various metabolism-related enzymes, and its modification sites are highly conserved with those of acetylation. However, compared with acetylation, succinylation induces a more significant charge reversal and a stronger steric hindrance effect; thus, it is more likely to cause substantial inhibition of enzyme activity and interference with protein-protein interactions, exerting a more critical impact on protein function (15). On one hand, the charge reversal effect arises from the conversion of the positively charged ϵ-amino group of lysine to a negatively charged succinyl group, which can significantly disrupt the electrostatic interaction network of proteins. For example, succinylation at lysine residues K122 of histone H3 and K77 of histone H4 impairs nucleosome stability, leading to defects in DNA damage repair and telomere silencing while enhancing gene transcription (16, 17). On the other hand, the steric hindrance effect of large molecular groups regulates protein activity through conformational rearrangement. For instance, OXCT1, acting as a novel succinyltransferase, catalyzes the modification of the K284 residue in the tumor suppressor protein LACTB. This modification inhibits LACTB’s protease activity, relieves the suppression of mitochondrial oxidative phosphorylation, and thereby promotes hepatocellular carcinoma progression (18). Succinylation is dynamically regulated not only through enzymatic reactions but also formed via non-enzymatic pathways (19). This modification is widely distributed in organelles such as mitochondria and the nucleus, as well as in cytoplasmic proteins, and is involved in regulating key biological processes including energy metabolism, redox homeostasis, and epigenetics (20). In cancer, succinylation influences tumor progression by modulating pathways such as glucose metabolism, histone modification, and redox homeostasis (11).

Succinylation, a pivotal post-translational modification (PTM), has been increasingly recognized in recent years as a crucial bridge linking metabolic reprogramming and immune regulation by reshaping protein function and stability (21). As the central hub of cellular energy metabolism, mitochondria exhibit a tight association between their function and succinylation—particularly, succinylation of key enzymes in the tricarboxylic acid (TCA) cycle has been shown to regulate mitochondrial metabolic flux and the production of reactive oxygen species (ROS) (22, 23). Recent studies have revealed that mitochondrial metabolic reprogramming regulates the activation and differentiation of immune cells via succinylation. For example, succinylation can alter the stability of HIF-1α, thereby perturbing the pro-inflammatory/anti-inflammatory polarization balance of macrophages and ultimately governing the ability of tumor cells to counteract anti-tumor immune responses (24, 25). Furthermore, succinate can also inhibit T cell effector function by suppressing the activity of succinyl-CoA synthetase and disrupting mitochondrial glucose oxidation (26).

Succinylation stands out among post-translational modifications as a distinctive regulatory layer in cancer immunotherapy precisely because it is predominantly metabolite-driven rather than enzyme-centric. Unlike phosphorylation, which functions as a rapid binary switch in signaling cascades, or acetylation and methylation, which primarily fine-tune epigenetic landscapes and enzyme activities through dedicated writer-eraser-reader systems, succinylation introduces a profound charge reversal (from +1 to -1) and significant steric hindrance via the bulky succinyl group, directly coupling TCA-cycle flux and succinyl-CoA availability to protein function (10, 27). This metabolic embedding allows succinylation to serve as a real-time sensor of the tumor microenvironment’s nutrient stress and Warburg-like reprogramming, enabling bidirectional control over immune checkpoints (e.g., PD-L1 stability), macrophage polarization, and T-cell exhaustion in ways that acetylation or ubiquitination achieve only indirectly. Consequently, succinylation not only reshapes metabolic networks but also translates these changes into immune outcomes more directly than other PTMs, offering unique therapeutic opportunities for integrating metabolic intervention with immunotherapy (28).

This review will systematically elaborate on the molecular mechanisms, biomarker screening, and therapeutic intervention strategies of succinylation in cancer immunotherapy. It aims to provide a theoretical framework for in-depth dissection of the “succinylation–metabolic reprogramming–immune regulation” crosstalk network, facilitate the development of precision therapeutic strategies based on succinylation modulation, promote the clinical translational integration of metabolic drugs and immunotherapies, and ultimately achieve a paradigm shift in cancer treatment from “single-targeted therapy” to “systematic intervention”.

## 2 Regulatory framework of succinylation

### 2.1 Regulatory enzyme systems

**Figure 2:**
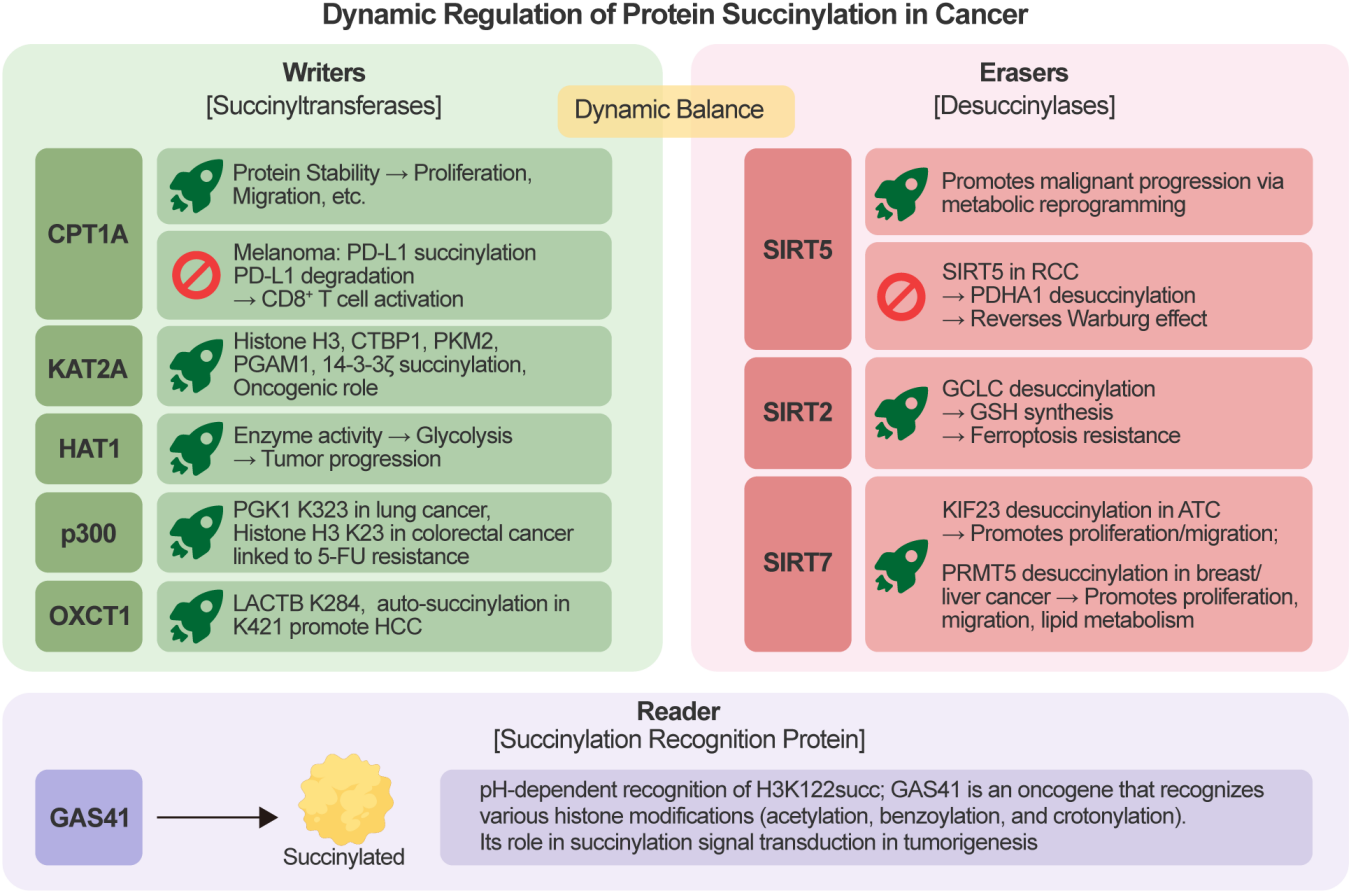
Regulatory mechanisms of succinylation in cancer: reported enzymes and context-dependent recognition. Protein succinylation levels are influenced by metabolic availability of succinyl-CoA and a limited set of reported enzymes that promote (putative writers, e.g., CPT1A, KAT2A, HAT1, p300, OXCT1) or remove (erasers, mainly SIRT2, SIRT5, SIRT7) the modification in context-dependent manners. Only one reader protein has been reported to date: GAS41, which specifically recognizes histone H3 lysine 122 succinylation (H3K122succ) in a pH-dependent manner within chromatin contexts; its recognition of succinylation is currently validated only for this histone site and condition, and no broad or global succinylation reader system has been established. CPT1A, Carnitine palmitoyltransferase 1A; KAT2A, Lysine acetyltransferase 2A; HAT1, Histone acetyltransferase 1; p300, E1A-binding protein p300; OXCT1, 3-oxoacid CoA-transferase 1; SIRT5, Sirtuin 5; RCC, Renal cell carcinoma; PDHA1, Pyruvate dehydrogenase E1α subunit; SIRT2, Sirtuin 2; GCLC, Glutamate-cysteine ligase catalytic subunit; GSH, Glutathione; SIRT7, Sirtuin 7; ATC, Anaplastic thyroid cancer; PRMT5, Protein arginine methyltransferase 5; GAS41, Glioma amplified sequence 41; H3K122succ, Histone H3 lysine 122 succinylation; HCC, Hepatocellular carcinoma. Created by Figdraw.

Unlike classical post-translational modifications such as acetylation or methylation, lysine succinylation is predominantly driven by intracellular metabolic states, particularly the availability of succinyl-CoA. Although a regulatory framework involving “writers,” “erasers,” and “readers” has been proposed, this architecture remains incompletely defined and appears to be highly context-dependent (29, 30) (**Figure 2**). Among these, Writers (i.e., succinyltransferases) catalyze the transfer of succinyl groups to substrate proteins, while Erasers (i.e., desuccinylases) mediate the enzymatic removal of succinylation modifications (**Table 1**).

**Table 1:** Regulatory enzyme systems of succinylation.

Enzyme function	Enzyme	Substrate	Sites	Regulation of cancer	References
Writers	CPT1A	SP5	K391	Promotion	(35)
		S100A10	K47	Promotion	(36)
		MFF	K302	Promotion	(37, 38)
		LDHA	K318, K222	Promotion	(39, 40)
		14–3–3θ	K85	Promotion	(41)
		PD-L1	K129	Suppression	(42)
	KAT2A	Histone H3	K79	Promotion	(43)
		CTBP1	K46, K280	Promotion	(44)
		PKM2	K475	Promotion	(45)
		PGAM1	K161	Promotion	(46)
		14-3-3ζ	K79	Promotion	(47)
	HAT1	PGAM1	K99	Promotion	(48)
	p300	PGK1	K323	Promotion	(33)
		Histone H3	K23	Promotion	(49)
		GCLC	K38, K126, K326	Suppression	(52)
	LACTB	LACTB	K284	Promotion	(18)
Erasers	SIRT2	GCLC	K38, K126, K326	Promotion	(52)
	SIRT5	ME2	K346	Promotion	(53)
		PPA2	K176	Promotion	(54)
		CS	K393, K395	Promotion	(55)
		SHMT2	K280	Promotion	(56)
		BCAT1	K39	Promotion	(57)
		SDHA	K547	Promotion	(58)
		PKM2	K498	Promotion	(59)
		IDH2	K413	Promotion	(60)
		c-myc	K369	Promotion	(61)
		PDHA1	K351	Suppression	(62)
	SIRT7	KIF23	K537	Promotion	(63)
		PRMT5	K387	Promotion	(64)

Compared with well-characterized post-translational modifications such as acetylation, the concept of “reader” proteins for lysine succinylation is still in its infancy. To date, GAS41 is the only protein reported to recognize succinylated histones; however, whether it functions as a bona fide “reader” analogous to those in other PTM systems remains to be fully established (31). Importantly, this recognition appears to be highly site-specific and restricted to a histone context, and current evidence does not support a generalized role for GAS41 as a global succinylation reader. Whether GAS41 or other proteins can recognize non-histone succinylation marks, or function broadly across different cellular contexts, remains to be determined. At the pan-cancer level, GAS41 promotes precise regulation of cell growth and development by recognizing key histone modifications (e.g., acetylation, benzoylation, and crotonylation), thereby functioning as an oncogenic factor (32). However, the role of GAS41-regulated succinylation signal transduction in modulating tumor initiation and progression remains unclear.

It should be noted that, in contrast to canonical acetyltransferases, many reported “succinyltransferases” may not function as dedicated enzymes but rather facilitate or enhance succinylation under specific metabolic conditions. In mammals, multiple proteins have been reported to promote succinylation of specific substrates in distinct experimental contexts; these include carnitine palmitoyltransferase 1A (CPT1A), lysine acetyltransferase 2A (KAT2A/GCN5), histone acetyltransferase 1 (HAT1), E1A-binding protein p300 (p300), and 3-oxoacid CoA-transferase 1 (OXCT1) (11, 18, 33), but they should not be uniformly classified as canonical, substrate-specific succinyltransferases (i.e., bona fide “Writers”) without qualification. Notably, these proteins lack well-characterized succinyltransferase domains that are conserved in classical PTM writers (34), and their succinyltransferase activity is often context-dependent, with mechanistic details remaining incompletely elucidated. For instance, CPT1A, a central node in metabolic regulation, undergoes autosuccinylation and has been shown to catalyze succinylation of specific substrates (including SP5 (35), S100A10 (36), MFF (37, 38), LDHA (39, 40) and 14-3-3θ (41)) in prostate, gastric, ovarian cancers, and lymphoma, thereby stabilizing these proteins, enhancing proliferation, migration, invasion, and stemness, and ultimately fueling tumorigenesis; however, its primary physiological function is as a key enzyme in mitochondrial fatty acid oxidation, and its succinyltransferase activity is secondary and context-specific rather than a canonical, dedicated function. Notably, CPT1A’s effect on protein stability is context-dependent: in melanoma, CPT1A-mediated succinylation of PD-L1 triggers its proteasomal degradation, boosts CD8+ T-cell activation, and suppresses tumor growth (42). KAT2A acts as an oncoprotein in glioma, prostate, gastric, liver, and pancreatic cancers by succinylating histone H3 (43), CTBP1 (44), PKM2 (45), PGAM1 (46) and 14-3-3ζ (47), thereby driving proliferative and aggressive phenotypes. HAT1 is markedly upregulated in hepatocellular, pancreatic, and biliary cancers, where it succinylates PGAM1 at K99, augments glycolytic flux, and accelerates tumor growth both *in vitro* and *in vivo* (48). In lung cancer, p300-catalyzed succinylation of PGK1 at K323 reinforces glycolysis and metabolic rewiring, promoting malignant progression (33). In colorectal cancer, p300-dependent H3K23 succinylation emerges as a key epigenetic driver of 5-fluorouracil (5-FU) resistance (49). Finally, the ketone body-metabolizing enzyme OXCT1 moonlights as a succinyltransferase: it promotes hepatocellular carcinoma by succinylating LACTB at K284 (18), while autosuccinylation at its own K421 enhances ketolytic metabolism and tumor growth (50).

The dynamic regulation of succinylation depends on the action of desuccinylases, primarily members of the NAD^-^-dependent deacetylase (sirtuin) family—specifically SIRT2, SIRT5, and SIRT7. These enzymes play key roles in metabolic regulation, stress responses, aging, and disease (51). As a desuccinylase, SIRT2 catalyzes the desuccinylation of glutamate-cysteine ligase catalytic subunit (GCLC) at residues K38, K126, and K326. In renal cell carcinoma (RCC) and osteosarcoma cells, this desuccinylation promotes glutathione (GSH) synthesis, thereby enhancing cancer cell resistance to ferroptosis. In contrast, succinylation of GCLC mediated by the succinyltransferase p300 increases cancer cell susceptibility to ferroptosis (52). In colorectal cancer, SIRT5-mediated desuccinylation of malic enzyme 2 (ME2) enhances mitochondrial respiration to promote tumor cell growth (53); its mediated desuccinylation of inorganic pyrophosphatase 2 (PPA2) increases the stability of HIF-1α under hypoxic conditions, thereby facilitating tumor cell metastasis (54); and its mediated desuccinylation of citrate synthase (CS) promotes the proliferation and migration of colorectal cancer cells (55). Furthermore, in osteosarcoma, glioma, RCC, lung cancer, and chordoma, SIRT5 catalyzes the desuccinylation of SHMT2 (56), BCAT1 (57), SDHA (58), PKM2 (59), IDH2 (60) and c-Myc (61). SIRT5 facilitates malignant tumor progression by driving multiple metabolic reprogramming processes, including serine catabolism, the inhibition of cancer cell sensitivity to ferroptosis, and the promotion of antioxidant responses. Notably, however, in RCC, the low succinylation state of pyruvate dehydrogenase E1α subunit (PDHA1) induced by SIRT5 exerts a tumor-suppressive effect by reversing the Warburg effect (62). Another desuccinylase, SIRT7, promotes the proliferation and migration of anaplastic thyroid cancer cells by regulating the desuccinylation of kinesin family member 23 (KIF23) (63). In breast cancer and liver cancer cells, SIRT7 catalyzes the desuccinylation of protein arginine methyltransferase 5 (PRMT5), which in turn promotes lipid metabolic reprogramming, tumor growth, and metastasis (64). Collectively, current evidence suggests that the regulatory landscape of succinylation is less hierarchically organized than that of classical PTMs and is more tightly coupled to metabolic flux and microenvironmental conditions.

### 2.2 Metabolic networks regulated by succinylation

Succinylation is a covalent modification process driven by succinyl-CoA, whose core mechanism relies on the dynamic balance of mitochondrial metabolic intermediates. As the primary site for succinyl-CoA synthesis, mitochondria’s related metabolic pathways directly determine the global level of intracellular succinylation by regulating the production rate of succinyl-CoA (10). The tricarboxylic acid (TCA) cycle is the core pathway of energy metabolism in aerobic organisms, and it also provides a common pathway and metabolic intermediates for glucose, lipid, and amino acid metabolism. In the TCA cycle, α-ketoglutarate (α-KG) is converted to succinyl-CoA under the catalysis of the α-ketoglutarate dehydrogenase complex (KGDHC). Thus, KGDHC directly influences the succinylation process by regulating succinyl-CoA levels (65, 66). For example, in yeast, inducing the expression of E1k (a subunit of KGDHC) increases the cellular succinylation level by approximately 1.7-fold, while E1k gene deletion reduces it to around 25% (15). Isocitrate dehydrogenase (IDH) is a key enzyme that catalyzes the oxidative decarboxylation of isocitrate to generate α-KG in the TCA cycle. Its family is classified into NADP^-^-dependent (IDH1 and IDH2) and NAD^-^-dependent (IDH3) subtypes. In glioma, IDH1 mutation can induce a hyper-succinylation state, which in turn leads to respiratory inhibition, induces mitochondrial depolarization, aggravates cancer metabolic reprogramming, promotes BCL-2 accumulation, and enhances tumor cell survival (67). Succinate dehydrogenase (SDH), also known as mitochondrial Complex II, is a heterotetramer composed of four subunits (SDHA, SDHB, SDHC, and SDHD) and is responsible for converting succinate to fumarate in the TCA cycle (68). Germline mutations in SDH subunits are associated with tumors such as paraganglioma and renal cell carcinoma (RCC); these mutations inactivate the SDH complex and cause succinate accumulation. Accumulated succinate can inhibit the activity of α-KG-dependent enzymes and induce abnormal protein modifications via succinyl-CoA accumulation, collectively exacerbating metabolic disorders and tumor progression (69). Additionally, compared with other mutant tumors, IFN-γ-induced gene expression is severely suppressed in SDH-deficient tumors, indicating that succinate may also influence tumor progression by regulating anti-tumor immune responses *in vivo* (26).

Glycolysis serves as the core pathway for energy metabolism in tumor cells. Even under oxygen-sufficient conditions, tumor cells remain highly dependent on glycolysis to meet the demands of rapid proliferation and microenvironmental adaptation—a phenomenon known as the Warburg effect (70). Pyruvate Kinase (PK) is a key rate-limiting enzyme in the glycolytic pathway; tumor cells tend to highly express the embryonic isoform PKM2, which leads to the accumulation of glycolytic intermediates (71). The succinyltransferase KAT2A drives gastric cancer progression by promoting succinylation at the K475 site of PKM2, which inhibits PKM2 activity (45). Similarly, KAT3B (p300) mediates succinylation at the K298 site of PKM2 to promote glycolysis and the malignant progression of lung cancer (72). In contrast, SIRT5 deficiency induces excessive succinylation of PKM2, which in turn regulates the metabolic remodeling of macrophages (73, 74), eosinophils (75), and tumor cells (59). Phosphoglycerate Mutase 1 (PGAM1) is another key enzyme in glycolysis: HAT1 can catalyze succinylation at the K99 site of PGAM1, which enhances its enzymatic activity, stimulates glycolytic flux in cancer cells, and ultimately promotes tumorigenesis (48, 76). Deletion of p300 leads to a significant reduction in the succinylation levels of multiple glycolytic enzymes, including Phosphoglycerate Kinase 1 (PGK1), and mutations at the succinylation sites of PGK1 markedly impede cellular glycolysis (33, 77). Similarly, the expression of P4HA1 enhances succinylation at the K191 and K192 sites of PGK1 by increasing succinate concentration; this modification inhibits the proteasomal degradation of PGK1, thereby significantly increasing aerobic glycolysis (25). Furthermore, CPT1A induces succinylation at the K318 and K222 sites of Lactate Dehydrogenase A (LDHA), a key enzyme in the Warburg effect, increasing LDHA stability and protein level to exert a pro-tumorigenic role in lymphoma and gastric cancer (39, 40). Thus, succinylation profoundly influences the Warburg effect in tumor cells by regulating the activity of key glycolytic enzymes such as PKM2, PGAM1, PGK1, and LDHA.

In addition to glucose metabolism, lysine succinylation impacts tumor progression via multiple routes, such as modulating amino acid metabolic reprogramming, sustaining lipid metabolic homeostasis, and regulating redox balance. These interrelated metabolic pathways jointly constitute a highly integrated regulatory network that exerts a significant impact on the clinical prognosis of patients with tumors. Glutaminase (GLS) serves as a key enzyme catalyzing the breakdown of glutamine to glutamate, and it fulfills a core function in amino acid metabolism, energy provision, and biosynthesis (78). In pancreatic ductal adenocarcinoma (PDAC), GLS promotes tumor cell proliferation, and the succinylation level of GLS at residue K311 exhibits a positive correlation with unfavorable prognosis in PDAC patients (79). Protein arginine methyltransferase 5 (PRMT5) modulates the expression of critical genes involved in lipid metabolic pathways by mediating arginine methylation of histone and non-histone proteins. Desuccinylation of PRMT5 at the K387 site facilitates lipid metabolic reprogramming, tumor growth, and *in vitro* and *in vivo* metastasis (64). Copper-zinc superoxide dismutase (SOD1) is a key antioxidant enzyme; the loss of SOD1 is linked to multiple diseases, cancer included. Research has shown that succinylation of SOD1 impairs its activity, thus facilitating tumor progression (80). Moreover, desuccinylation of PKM2 and IDH2 can also strengthen the antioxidant activity of tumor cells and drive tumor growth (59, 60).

**Figure 3:**
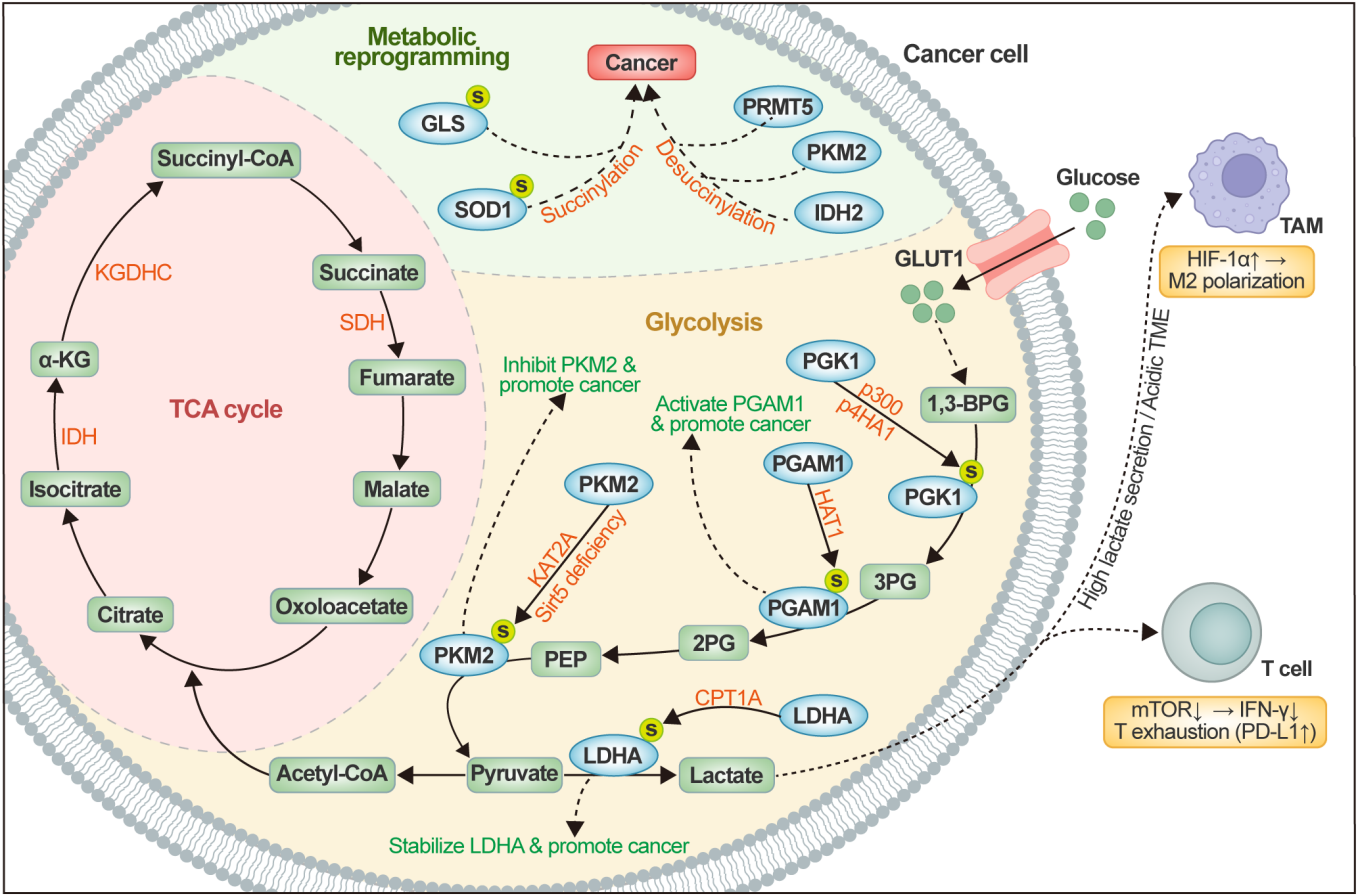
Succinylation-mediated metabolic reprogramming and immune crosstalk in the tumor microenvironment. Succinyl-CoA, generated via the TCA cycle’s α-ketoglutarate dehydrogenase complex (KGDHC), serves as the primary substrate for lysine succinylation, modulating key glycolytic enzymes (e.g., PKM2, PGAM1, PGK1, LDHA) and metabolic proteins (e.g., GLS, SOD1, PRMT5) through writer- and eraser-mediated modifications. These alterations promote tumor-intrinsic metabolic reprogramming, including enhanced Warburg effect and lactate overproduction. The resulting high lactate secretion creates an acidic tumor microenvironment that directly influences adjacent immune cells: in tumor-associated macrophages, elevated lactate stabilizes HIF-1α, driving M2-like polarization with increased Arg1 and IL-10 expression while suppressing antigen presentation; in CD8^-^ T cells, lactate impairs mTOR signaling, reduces IFN-γ production, and promotes exhaustion phenotypes (PD-1^-^TIM-3^-^). Conversely, succinylation-dependent degradation of PD-L1 via CPT1A in tumor cells can relieve checkpoint inhibition on T cells. This diagram illustrates the bidirectional succinylation-mediated crosstalk between tumor metabolic rewiring and immune cell functional reprogramming. KGDHC, α-Ketoglutarate dehydrogenase complex; α-KG, α-Ketoglutarate; IDH, Isocitrate dehydrogenase; SDH, Succinate dehydrogenase; TCA cycle, Tricarboxylic acid cycle; PKM2, Pyruvate kinase M2; KAT2A, Lysine acetyltransferase 2A; SIRT5, Sirtuin 5; PEP, Phosphoenolpyruvate; 2PG, 2-Phosphoglycerate; PGAM1, Phosphoglycerate mutase 1; 3PG, 3-Phosphoglycerate; PGK1, Phosphoglycerate kinase 1; 1,3-BPG, 1,3-Bisphosphoglycerate; CPT1A, Carnitine palmitoyltransferase 1A; LDHA, Lactate dehydrogenase A; GLUT1, Glucose transporter 1; GLS, Glutaminase; SOD1, Copper-zinc superoxide dismutase; PRMT5, Protein arginine methyltransferase 5; IDH2, Isocitrate dehydrogenase 2.

Functioning as a succinyl-CoA-dependent dynamic post-translational modification, lysine succinylation significantly remodels the metabolic network of tumor cells by targeting core enzymes in critical pathways such as the tricarboxylic acid (TCA) cycle, glycolysis, amino acid metabolism, and redox balance (**Figure 3**). In the TCA cycle, succinyl-CoA production is tightly regulated by enzyme complexes like KGDHC, IDH, and SDH, and variations in its concentration has been shown to dictate the level of global succinylation. In the glycolytic pathway, succinylation modulates rate-limiting enzymes (e.g., PKM2, PGAM1, PGK1, and LDHA) to has been shown to contribute to strengthen the Warburg effect and sustain glucose metabolic reprogramming (81). Additionally, this modification also extensively infiltrates interconnected metabolic networks. These interactions together constitute a highly integrated metabolic regulatory network; its imbalance not only fulfills the proliferation and survival requirements of tumor cells but also shows a significant association with adverse patient prognosis, underscoring the pivotal role of succinylation in metabolic adaptation.

## 3 Role of succinylation in the immune regulatory system

### 3.1 Why succinylation constitutes a distinctive regulatory layer in cancer immunotherapy

Compared with other well-studied post-translational modifications, lysine succinylation occupies a unique position at the metabolism-immunity interface. Phosphorylation and ubiquitination primarily govern signal transduction and protein turnover, respectively, while acetylation modulates both epigenetics and metabolic enzymes through highly evolved enzymatic cascades. Succinylation, however, is intrinsically tied to mitochondrial succinyl-CoA levels, rendering it exquisitely sensitive to the metabolic vulnerabilities of the tumor microenvironment—such as hypoxia-driven TCA rewiring and lactate accumulation. This direct metabolic linkage, combined with its stronger electrostatic and steric effects on target proteins, allows succinylation to exert dual, context-dependent influences on anti-tumor immunity (from PD-L1 degradation to lactate-mediated immunosuppression) that transcend the scope of purely transcriptional or degradative PTMs, thereby providing a conceptual framework for precision metabolic-immunotherapeutic strategies (18, 42).

### 3.2 Direct regulation of immune checkpoints

Immune Checkpoint Blockade (ICB) therapy, a landmark breakthrough in cancer immunotherapy, reshapes anti-tumor immune responses by relieving T cell inhibitory signals, significantly improving clinical outcomes for patients with various advanced malignancies (82). However, its efficacy exhibits marked tumor-type and inter-individual heterogeneity, with primary/acquired resistance being widespread (83). Current optimization strategies adopt a two−pronged approach: precise biomarkers such as PD-L1 expression, tumor mutational burden (TMB), and microsatellite instability (MSI) are utilized for patient stratification to reduce ineffective treatments and immune-related adverse events (irAEs), while in-depth dissection of resistance mechanisms including T cell exhaustion, an immunosuppressive microenvironment, and defective antigen presentation informs the design of targeted combination therapies to overcome resistance (84–86).

Notably, the crosstalk between metabolic reprogramming and immune evasion is emerging as a new paradigm for overcoming the bottleneck of ICB resistance. Recent studies have revealed that CPT1A-a key enzyme in mitochondrial fatty acid oxidation-can catalyze the succinylation of PD-L1 at the K129 site, triggering its ubiquitin-dependent degradation pathway and significantly accelerating PD-L1 clearance via the endosome-lysosome system. This process directly impairs the immunosuppressive “molecular shield” function of surface PD-L1 on tumor cells, reverses the dysfunctional state of CD8+ T cells, and thereby enhances anti-tumor immune surveillance. These studies have demonstrated that CPT1A-mediated succinylation of PD-L1 at K129 can trigger its ubiquitin-dependent degradation and lysosomal clearance in melanoma and other models, thereby contributing to reduced surface PD-L1 levels, relief of CD8^+^ T cell inhibition, and enhanced anti-tumor responses. This process links lipid metabolic reprogramming with immune checkpoint regulation in a context-dependent manner and offers a potential target for metabolic intervention to improve immunotherapy efficacy. The mechanism has been reported in several high-metabolism solid tumors such as melanoma, NSCLC, and colorectal cancer, suggesting that modulation of succinylation may serve as a sensitization approach in these specific contexts rather than a universal strategy (42).

### 3.3 Regulating immunity via mediating macrophage polarization

Macrophages exert dual functions in anti-tumor immunity: they activate T cells by presenting tumor antigens through MHC-I/II molecules, while simultaneously having their function suppressed by the tumor microenvironment (TME)—a process that weakens immune responses. Specifically, pro-inflammatory M1 macrophages are capable of directly killing tumor cells, whereas anti-inflammatory M2 macrophages facilitate immune evasion, stimulate angiogenesis, and foster the development of an immunosuppressive microenvironment, all of which contribute to tumor progression. Targeting macrophage polarization (such as enhancing M1 polarization or suppressing M2 polarization) has emerged as a critical strategy to reverse immunosuppression (87). Succinate, a metabolic messenger in innate immunity, drives inflammatory reactions via the HIF-1α/IL-1β axis: its accumulation triggers HIF-1α-dependent expression of IL-1β (a hallmark cytokine of M1 macrophages), solidifying its status as a central inflammatory signal (88). The desuccinylase SIRT5 precisely modulates macrophage polarization by regulating the succinylation state of metabolic enzymes. For one thing, loss of SIRT5 causes excessive succinylation of PKM2, which promotes a shift in PKM2’s active conformation from a tetramer to a dimer and reduces its pyruvate kinase activity. This alteration boosts glycolytic flux, substantially elevates IL-1β production, induces M1 polarization, and amplifies inflammatory responses (73). For another, SIRT5-mediated desuccinylation of TBK1 exerts a negative regulatory effect on inflammatory signaling in macrophages, and its ability to inhibit excessive inflammation has been validated in sepsis models (89). These mechanisms collectively indicate that SIRT5 serves as a pivotal target connecting metabolic reprogramming and immune polarization.

Recent research has demonstrated that succinate-loaded tumor-derived microparticles (SMPs) are capable of remodeling the metabolic state of tumor-associated macrophages (TAMs). By delivering succinate to mitochondria and the nucleus, SMPs induce succinylation of histone H3K122 in the promoter regions of isocitrate dehydrogenase 2 (IDH2) and lactate dehydrogenase A (LDHA). This leads to enhanced glycolysis and reduced tricarboxylic acid (TCA) cycle activity, which in turn promotes classical M1-like polarization of macrophages and ultimately activates anti-tumor immune responses (90). In contrast, within the TME, α-ketoglutarate (α-KG) modulates macrophage polarization through regulating energy metabolism in the TCA cycle—a process that reprograms macrophages from an M1 to an M2 phenotype. This compromise in their antigen-presenting ability results in the suppression of T cell-mediated immune responses (91). In advanced cholangiocarcinoma, succinylation of pyruvate dehydrogenase E1α subunit (PDHA1) at residue K83 modifies the enzyme’s activity and modulates metabolic flux, resulting in the accumulation of α-KG in the TME. Activation of the OXGR1 receptor on macrophages by this accumulated α-KG triggers mitogen-activated protein kinase (MAPK) signaling and suppresses MHC-II antigen presentation, ultimately facilitating immune evasion and tumor progression (92). Notably, PDHA1 is also a target for metabolic intervention in other cancers: PIK-III, a glycolysis inhibitor, re-sensitizes renal cell carcinoma to cuproptosis by activating PDHA1 via thiamine metabolism reprogramming, underscoring the broad therapeutic potential of targeting PDHA1 modifications (93).

### 3.4 Mediating dendritic cell differentiation to regulate immunity

The differentiation status of dendritic cells (DCs) plays a central role in tumor immune regulation. Under physiological conditions, DCs activate tumor-specific T cell responses through antigen presentation and the expression of costimulatory molecules (CD80/CD86). However, in the tumor microenvironment (TME), immunosuppressive factors induce DCs to differentiate into regulatory DCs—defined by downregulated costimulatory molecules, upregulated immune checkpoint molecules (e.g., PD-L1), and increased expression of immunosuppressive enzymes (IDO, ARG1). This phenotypic transition deprives DCs of their ability to activate T cells; instead, it promotes the expansion of regulatory T cells and the suppression of effector T cell function, thereby shaping an immune-evasive microenvironment (94, 95). Recent studies have shown that targeting key pathways involved in DC differentiation (e.g., the FLT3L/Flt3 signaling axis) or combining with immune checkpoint blockade therapy can reverse the immunosuppressive phenotype of DCs, providing a new strategy for cancer immunotherapy (96). Notably, succinyl-CoA ligase β subunit (Suclg2), a key metabolic enzyme, exhibits significantly higher expression in regulatory DCs than in mature DCs. This enzyme maintains the immunosuppressive properties of DCs by inhibiting the succinylation modification of Lactb at the K288 site, which in turn downregulates the activity of the NF-κB signaling pathway. This finding uncovers a novel mechanism by which metabolic enzymes regulate DC function via succinylation modification (97).

### 3.5 Rewiring adaptive immunity through succinylation

#### 3.5.1 Regulating T cell metabolism and functional exhaustion

In the tumor microenvironment (TME), metabolic reprogramming and functional exhaustion of T cells serve as core causes of immune response failure. Metabolic stress (e.g., glucose competition, lipid peroxidation) directly inhibits CD8^-^ T cell activity. For example, cancer cells consume glucose and accumulate lactate via the Warburg effect, which suppresses mTOR activity and IFN-γ production in T cells; meanwhile, CD36-mediated uptake of oxidized lipids induces ferroptosis in CD8^-^ T cells, and GPX4 deficiency further exacerbates their functional exhaustion (98, 99). In anti-tumor immunity, the functional exhaustion of CD8^-^ T cells is closely associated with PD-L1 expression in tumor cells. Studies have shown that succinyl-CoA can drive succinylation of PD-L1 at the K129 site via CPT1A catalysis, triggering PD-L1 degradation through the endosome-lysosome pathway. This process reduces PD-L1 expression on the tumor cell surface, relieves its inhibitory effect on CD8^-^ T cells, and thereby enhances T cell cytotoxicity and anti-tumor responses. Conversely, decreased CPT1A activity leads to PD-L1 accumulation, promoting T cell exhaustion (42). Furthermore, succinylation irreversibly disrupts T cell metabolic homeostasis by reprogramming the BCAA-fatty acid metabolic axis, which acts as a key driver of their terminal exhaustion. Targeting this pathway (e.g., enhancing SIRT7 activity or inhibiting BCAA metabolism) is expected to reverse T cell dysfunction, providing a new target for cancer immunotherapy (100).

#### 3.5.2 Modulation of regulatory T cell function

Treg cells function to suppress the activation of effector T cells (e.g., Th1, Th17 subsets), dendritic cells, and macrophages—either by secreting anti-inflammatory cytokines (including IL-10, TGF-β, IL-35) or through direct cell-cell interactions (e.g., via the CTLA-4 or PD-1 pathways) (101). Consequently, during infection or tissue damage, Treg cells restrict inflammatory responses and protect tissue homeostasis; in the TME, however, their overactivation inhibits anti-tumor immunity and facilitates immune evasion (102). Research has demonstrated that in inflammatory bowel disease (IBD), succinate accumulation impacts Treg cell function by regulating the post-translational modification of FOXP3, thereby perturbing immune homeostasis. Increased succinate levels downregulate OGDHc expression, which reduces the succinylation of FOXP3 protein. This leads to the exposure of FOXP3’s lysine residues, triggering ubiquitination-mediated degradation and ultimately reducing FOXP3 stability. As FOXP3 is a pivotal transcription factor for Treg cells, decreased FOXP3 expression weakens the immunosuppressive capacity of Treg cells and simultaneously promotes the secretion of the pro-inflammatory cytokine IL-17, ultimately aggravating intestinal inflammation. This study uncovers the essential role of FOXP3 succinylation in sustaining Treg cell function, elucidates the molecular mechanism by which succinate modulates immune homeostasis via the OGDHc-FOXP3 succinylation axis, and offers a novel therapeutic target for autoimmune disorders like IBD (103).

### 3.6 Regulating immunity by mediating intrinsic tumor cell traits

#### 3.6.1 Sustaining the stemness of cancer stem cells

Stemness in cells drives tumor immune evasion via multiple mechanisms: For one, CSCs have low expression of MHC-I molecules and tumor-associated antigens, lowering their likelihood of being recognized by T cells and NK cells. For another, they secrete immunosuppressive factors (e.g., TGF-β, IL-10), recruit regulatory T cells (Tregs) and myeloid-derived suppressor cells (MDSCs), and overexpress immune checkpoint molecules such as PD-L1 and CD47—all of which suppress the function of effector immune cells (104). Furthermore, CSCs remodel an immunosuppressive microenvironment through metabolic reprogramming, which further weakens anti-tumor immune responses. These characteristics collectively equip CSCs with immune evasion capacity, facilitating tumor progression and resistance to therapy (105). Importantly, the succinyltransferase CPT1A is capable of sustaining the stemness of ovarian cancer cells: it catalyzes the succinylation of mitochondrial fission factor (MFF) to reduce cellular lipid saturation. This process not only maintains stem cell properties but also increases the sensitivity of cancer cells to paclitaxel/cisplatin (38).

#### 3.6.2 Facilitating immunogenic cell death: pyroptosis

By releasing pro-inflammatory cytokines (IL-1β, IL-18) and damage-associated molecular patterns (DAMPs), pyroptosis activates dendritic cells (DCs) and facilitates CD8^-^ T cell infiltration, thereby boosting adaptive anti-tumor immune responses (106). Specifically, the pyroptosis executor protein GSDME is cleaved by Caspase-3 to induce pore formation in the plasma membrane. This event directly triggers tumor cell pyroptosis, potentiates the immunogenic cell death (ICD) effect, and drives the transition from “cold tumors” to “hot tumors,” which substantially increases responsiveness to immunotherapeutic interventions (107, 108). Consequently, GSDME-dependent pyroptosis acts as a pivotal link between chemotherapy/radiotherapy and immunotherapy, as it enhances tumor immunogenicity and T cell activation. In head and neck squamous cell carcinoma (HNSCC), increased GSDME expression coordinates cisplatin-induced pyroptosis in cancer cells; importantly, succinyl-CoA-mediated succinylation of GSDME can directly promote its protein cleavage (without requiring Caspase-3 activation), ultimately improving anti-tumor immune efficacy by amplifying the ICD effect (109).

### 3.7 Shaping an immunosuppressive microenvironment by mediating metabolic reprogramming

Via the Warburg effect, tumor cells undergo metabolic reprogramming, which results in abnormally elevated glycolytic flux and substantial lactate generation. High lactate levels remodel the tumor immune microenvironment through a variety of mechanisms. This effect not only directly impairs the phagocytic activity of macrophages, the cytotoxic function of NK cells, and the activation potential of T cells, but also disrupts their anti-tumor capabilities by modulating surface molecules on immune cells; at the same time, lactate drives the expansion of regulatory T cells (Tregs), the activation of myeloid-derived suppressor cells (MDSCs), and the polarization of M2 macrophages—together creating an immunosuppressive microenvironment that promotes tumor immune evasion (110, 111). Succinylation, an important post-translational modification, exerts a critical regulatory role in tumor metabolic reprogramming, especially in the precise regulation of lactate biosynthesis pathways. This modification directly controls the activity and stability of several key enzymes in glycolysis, with its regulatory impact on Lactate Dehydrogenase A (LDHA) being most notable. Research has demonstrated that succinylation modification of LDHA markedly boosts its enzymatic activity, enhances the efficiency of pyruvate-to-lactate conversion, and thus greatly strengthens the lactate-producing ability of tumor cells (112). In glioblastoma (GBM), succinylation of PGK1 at residues K191 and K192 prevents PGK1 from being degraded by the proteasome, leading to a significant increase in aerobic glycolysis and subsequent high lactate levels—ultimately inducing immunosuppression in GBM cells (25). Furthermore, GRK2-mediated desuccinylation of PKM2 can decrease glycolysis in macrophages in the context of rheumatoid arthritis, ultimately suppressing the inflammatory phenotype of these macrophages (113).

Branched-chain amino acid (BCAA) accumulation enables the remodeling of glucose metabolism in CD8^-^ T cells, ultimately enhancing their effector functions and anti-tumor responses. In the tumor microenvironment (TME), however, competitive uptake of BCAAs by cancer cells and T cells frequently results in metabolic deficiency and functional exhaustion of CD8^-^ T cells (114). Targeting this metabolic pathway thus offers potential for reversing the immunosuppressive state of the immune microenvironment. Importantly, SIRT7 is predominantly overexpressed in T cells, and its depletion leads to excessive succinylation of the branched-chain α-ketoacid dehydrogenase complex (BCKDH), a core enzyme in BCAA catabolism, which in turn activates the BCAA catabolic pathway. This process facilitates acyl-CoA accumulation and fatty acid biosynthesis, suppresses IFN-γ production in T cells, and induces an exhausted phenotype (PD-1^-^TIM-3^-^), which significantly compromises anti-tumor immune function. This mechanism highlights that targeting SIRT7-mediated succinylation regulation represents a key strategy to reinstate the anti-tumor potency of T cells (100).

The Tumor Immune Microenvironment (TIME) constitutes a complex ecosystem encompassing diverse immune cells and regulatory factors, and exerts a decisive influence on tumor development, progression, and immunotherapeutic responses. Importantly, EGFR-mutant tumors display prominent immunosuppressive TIME traits, with a hallmark being a marked decrease in tumor-infiltrating lymphocytes (particularly effector CD8^-^ T cells); this is likely the key mechanism responsible for their suboptimal responsiveness to immune checkpoint inhibitors (ICIs) (115, 116). At the molecular mechanistic level, latest research has shown that SIRT5 boosts the enzymatic activity of acetyl-CoA acetyltransferase 1 (ACAT1) through mediating its desuccinylation, which in turn triggers the dissociation of the KEAP1-NRF2 complex. The released NRF2 translocates to the nucleus, where it binds to the antioxidant response element (ARE) within the promoter regions of target genes, leading to significant upregulation of genes including heme oxygenase-1 (HO-1). Meanwhile, this pathway suppresses the synthesis and secretion of chemokines CCL5 and CXCL10, ultimately compromising the chemotactic migration capacity and tumor infiltration efficacy of CD8^-^ T cells. This series of cascading reactions collectively reshapes the immunosuppressive TIME, offering a new mechanism to explain immunotherapy resistance (117).

Lysine succinylation displays a profound paradoxical duality in regulating tumor immunity: For one, it enhances host anti-tumor defenses through activating immunogenic cell death (e.g., enhancing pyroptosis via succinylation of GSDME), facilitating anti-inflammatory phenotypic transitions (e.g., Suclg2-mediated tolerogenic differentiation of DCs), and degrading immune checkpoints (e.g., PD-L1 degradation via the lysosomal pathway). For another, this modification paves the way for tumor immune evasion by sustaining cancer stem cell stemness (succinylation of MFF), driving immunosuppressive metabolism (LDHA succinylation facilitating lactate accumulation), and perturbing immune homeostasis (FOXP3 desuccinylation weakening Treg function). The core of this bidirectional regulation resides in the spatiotemporal specificity of modification sites under metabolic stress—succinylation functions as a “molecular code” for cellular metabolism, and how this code is “interpreted” relies on the synergistic interplay between metabolic substrate concentrations, modifier enzyme localization, and the functional context of target proteins within the microenvironment. This process ultimately forges the dynamic equilibrium between immune surveillance and immune evasion (118) (**Table 2**).

**Table 2:** Mechanism of succinylation in immune regulation system.

Regulatory mechanism in immune response	Substrate	Immunomodulatory effects	References
Directly regulates immune checkpoints	PD-L1 Succinylation	Immunostimulation	(42)
Mediates macrophage polarization to regulate immunity	IDH2, LDHA Succinylation	Immunostimulation	(90)
	PDHA1 Succinylation	Immunosuppression	(92)
Mediates metabolic reprogramming for immune regulation	PGK1 Succinylation	Immunosuppression	(25)
	Desuccinylation of Key Enzymes in the BCAA Catabolic Pathway	Immunostimulation	(100)
Mediates pyroptosis to regulate immunity	MFF Succinylation	Immunosuppression	(38)
Mediates pyroptosis to regulate immunity	GSDME Succinylation	Immunostimulation	(109)
Regulates the tumor immune microenvironment (TIME)	ACAT1 Desuccinylation	Immunosuppression	(117)
Mediates dendritic cell (DC) differentiation to regulate immunity	Lactb Desuccinylation	Immunosuppression	(97)
Mediates Treg cell activity for immune regulation	FOXP3 Succinylation	Immunostimulation	(103)

## 4 Clinical biomarkers of succinylation in cancer immunotherapy

Given the central role of succinylation in linking metabolic reprogramming and immune regulation, its potential as a source of clinically relevant biomarkers is increasingly recognized.

Emerging evidence suggests that lysine succinylation may hold potential as a source of clinical biomarkers in oncology, although current data remain largely exploratory. Given its tight coupling to mitochondrial metabolism and its impact on protein stability and function, alterations in succinylation patterns may reflect underlying metabolic states that are closely linked to tumor progression and immune regulation (81, 119).

At the prognostic level, global or protein-specific changes in succinylation have been reported to correlate with tumor aggressiveness and patient outcomes in certain cancer types, although these observations are primarily derived from preclinical or limited cohort studies. From a predictive perspective, mechanistic findings—such as CPT1A-mediated succinylation of PD-L1 influencing its degradation—raise the possibility that succinylation status may be associated with responses to immune checkpoint blockade (42), although direct clinical validation is still lacking. In addition, succinylation signatures may serve as indirect indicators of metabolic reprogramming within the tumor microenvironment, which is increasingly recognized as a determinant of immunotherapy efficacy.

Despite these promising observations, several limitations hinder the clinical translation of succinylation-based biomarkers. These include the lack of standardized detection platforms, insufficient large-scale clinical validation, and the context-dependent nature of succinylation dynamics. Future studies integrating quantitative proteomics with clinical datasets will be essential to determine whether succinylation can be reliably leveraged for patient stratification or therapeutic decision-making (120).

## 5 Therapeutic approaches targeting succinylation

**Figure 4:**
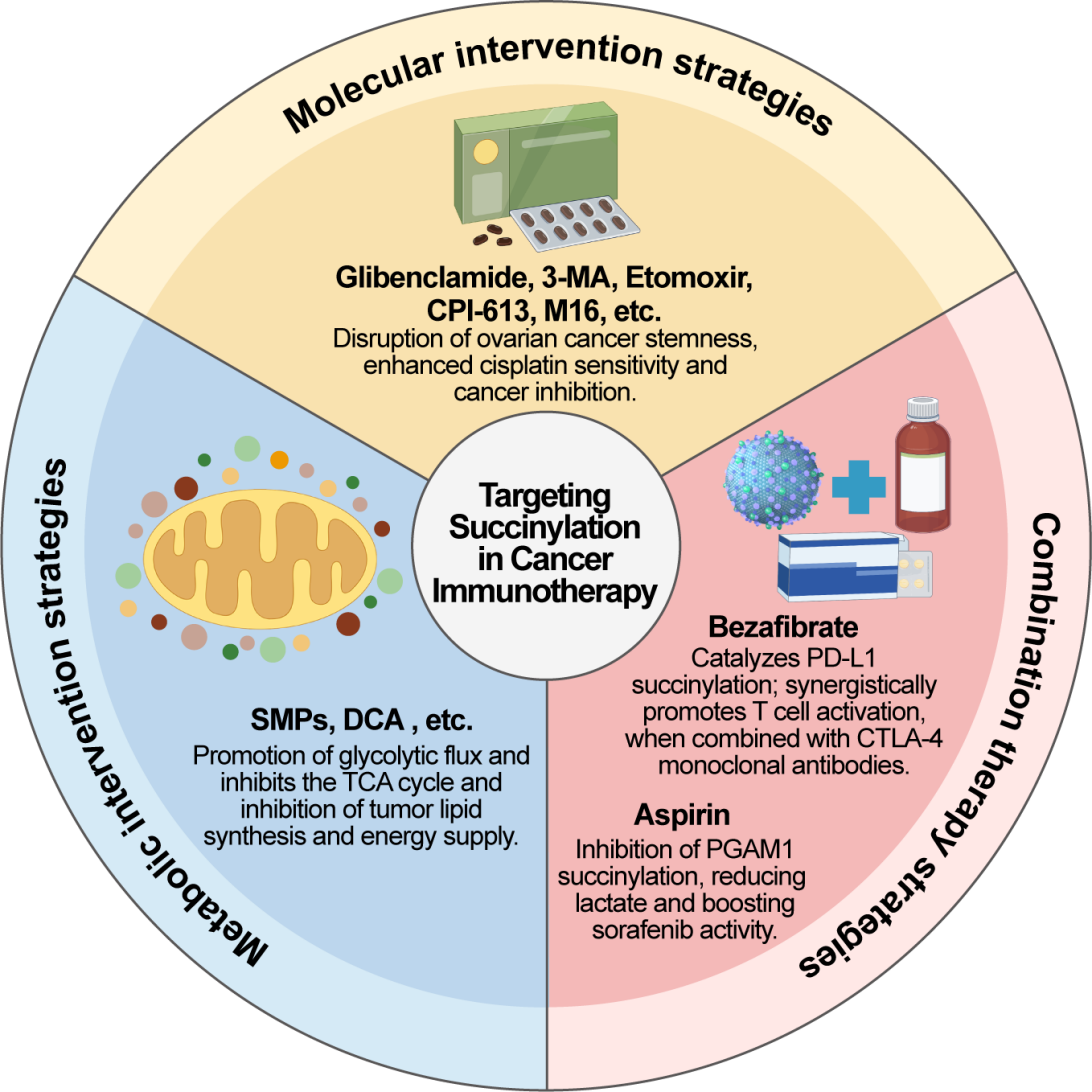
Therapeutic strategies targeting succinylation in cancer immunotherapy. This figure summarizes three strategies targeting succinylation for cancer immunotherapy: molecular interventions (e.g., Glibenclamide) disrupt cancer stem cell stemness; metabolic interventions (SMPs, DCA) and rewire metabolic flux to inhibit tumors; combination therapies (Bezafibrate^+^anti-CTLA-4, aspirin^+^sorafenib) synergistically enhance immune activation or treatment efficacy. SMPs, Succinate-loaded tumor-derived microparticles; DCA, Dichloroacetate; CTLA-4, Cytotoxic T-lymphocyte-associated protein 4; PGAM1, Phosphoglycerate mutase 1. Created by Figdraw.

Succinylation, a dynamic and reversible post-translational modification of proteins, profoundly participates in reshaping the tumor immune microenvironment by precisely modulating the activity of metabolic enzymes, signaling proteins, and epigenetic regulators. Its site-specific nature and dependence on metabolic substrates offer unique targets for interfering with tumor progression (**Table 3**). At the molecular intervention level, small-molecule agents exhibit considerable therapeutic potential (**Figure 4**).

**Table 3:** Anti-cancer agents targeting lysine succinylation/desuccinylation.

Compound	Mechanism	Cancer type	Ref.
Glyburide	Inhibits CPT1A’s LSTase activity → Blocks MFF succinylation → Disrupts cancer stemness	Ovarian cancer	(37)
3-MA	Inhibits PI3K → Suppresses CPT1A-mediated ATG16L1 succinylation → Inhibits autophagosome formation → Reverses cisplatin resistance	Hypopharyngeal carcinoma	(121)
Etomoxir	Inhibits CPT1A → Blocks ATG16L1 succinylation → Suppresses autophagy → Sensitizes to cisplatin	Hypopharyngeal carcinoma	(121)
CPI-613	Competitively inhibits PDHA1 K83 succinylation → Restores PDH activity → Blocks immune escape	Cholangiocarcinoma	(92)
Histidine	Activates SIRT5 → Promotes histone desuccinylation → Sensitizes to 6-mercaptopurine-induced apoptosis	Acute lymphoblastic leukemia	(122)
DK1-04e	Inhibits SIRT5 desuccinylase activity → Suppresses tumor growth	Breast cancer	(123)
M16	Inhibiting p23 succinylation →Suppresses COX-2 transcription	Lung adenocarcinoma	(124)
Succinate-loaded SMPs	Delivers succinate → Induces histone H3K122 succinylation → Drives TAMs toward M1 polarization	Solid tumors	(90)
BT2	Inhibits BCKDK → Reduces BCAA levels → Reverses SIRT7 deficiency-induced CD8^-^ T cell exhaustion	Colon cancer	(100)
Dichloroacetate (DCA)	Globally reprograms succinylation sites (179↑/114↓) → Inhibits metabolic enzymes → Blocks energy supply	Colorectal cancer	(125)
Bezafibrate (BEZ)	Activates PGC-1α → Upregulates CPT1A → Promotes PD-L1 succinylation degradation → Synergizes with anti-CTLA-4	Immunotherapy-resistant tumors	(42)
Aspirin	Inhibits HAT1 → Blocks PGAM1 K99 succinylation → Suppresses glycolysis → Enhances sorafenib efficacy	Hepatocellular carcinoma	(76)
Astragaloside IV (AS-IV)	Activates KAT2A → Promotes PGAM1 desuccinylation → Inhibits glycolysis and tumor viability	Hepatocellular carcinoma	(46)

For example, Glibenclamide specifically inhibits the lysine succinyltransferase (LSTase) activity of CPT1A, abrogates MFF succinylation, further impairs the stemness of ovarian cancer stem cells, and sensitizes tumors to cisplatin (37). Via a comparable mechanism, the PI3K inhibitor 3-MA suppresses assembly of the ATG12-ATG5-ATG16L1 complex, inhibits CPT1A-mediated succinylation of ATG16L1 and autophagosome biogenesis, and reverses cisplatin resistance. Moreover, Etomoxir, a CPT1A inhibitor, suppresses CPT1A’s enzymatic activity, blocks its succinylation of ATG16L1, disrupts autophagy activation, and directly reinstates cell sensitivity to cisplatin (121). All these studies validate CPT1A as a critical target for reversing chemoresistance. Furthermore, to target the immune evasion driven by PDHA1 K83 succinylation in cholangiocarcinoma, the small-molecule compound CPI-613 restores PDHA1’s enzymatic activity through competitive binding; when combined with gemcitabine/cisplatin, this regimen significantly improves the tumor inhibition rate (92). Importantly, activating protective desuccinylases also has clinical relevance: Histidine enhances the binding efficiency of SIRT5 to histone H3, facilitates histone desuccinylation, and markedly elevates the apoptotic response of relapsed/refractory leukemia cells to 6-mercaptopurine (122). In breast cancer, the selective SIRT5 inhibitor DK1-04e can markedly suppress breast tumor growth in tumor-bearing mouse models (123). In lung adenocarcinoma, researchers have screened M16, a potent succinylation inhibitor of the p23 protein; M16 effectively suppresses p23’s succinylation and nuclear translocation, reduces COX-2 transcription, and significantly restrains tumor growth (124).

Metabolic intervention approaches reshape the succinylation substrate pool to reverse the immunosuppressive microenvironment. A novel succinate-loaded tumor-derived microparticle (SMP) technique employs tumor cell membranes to encapsulate high-concentration succinate, achieving targeted delivery to macrophages through a membrane fusion mechanism and inducing succinylation at the H3K122 site of histones. This modification directly triggers the transcription of IDH2 and LDHA genes, promotes elevated glycolytic flux, suppresses the tricarboxylic acid (TCA) cycle, and eventually polarizes tumor-associated macrophages (TAMs) toward the pro-inflammatory M1 phenotype. This distinctive strategy employing cell membrane-derived microparticles loaded with endogenous metabolites for TAM polarization exhibits broad applicability in PTM-oriented tumor immunotherapies (90). Conversely, to address T cell exhaustion induced by the loss of the desuccinylase SIRT7, the BCKDK inhibitor BT2 decreases BCAA levels by inhibiting the phosphorylation of BCKDH. This reinstates mitochondrial respiratory function in CD8^-^ T cells and reverses the exhausted phenotype characterized by high PD-1 expression; importantly, BCAA-free dietary intervention yields comparable effects, offering a novel paradigm for metabolic diet-based therapy (100). Dichloroacetate (DCA) is a well-characterized PDK inhibitor. Research demonstrates that DCA globally reprograms 179 upregulated and 114 downregulated succinylation sites, specifically suppresses metabolic enzyme activity and molecular chaperone function, and directly impedes tumor lipid biosynthesis and energy supply. In this way, succinylation and acetylation establish a crosstalk network, which inhibits tumor proliferation and induces apoptosis through a “metabolism-PTM dual-axis” mechanism (125).

Combination therapeutic approaches further extend clinical translation potential. Functioning as the lysine succinyltransferase (LSTase) for PD-L1, CPT1A mediates PD-L1 succinylation and promotes its lysosome-dependent degradation, whereas CPT1A inhibition significantly increases PD-L1 protein abundance. The PGC-1α activator bezafibrate enhances CPT1A activity, thereby triggering PD-L1 succinylation and accelerating its lysosomal degradation. Its combination with anti-CTLA-4 monoclonal antibody (mAb) lowers PD-L1 expression, diminishes immune-related adverse events (irAEs), and exerts a synergistic effect on T cell activation (42). For sensitizing traditional treatments, aspirin suppresses the IKKβ/NF-κB p65 axis to abrogate HAT1-mediated PGAM1 succinylation at residue K99, which results in reduced lactate generation in HCC cells and substantially improves the therapeutic efficacy of Sorafenib (76). Astragaloside IV (AS-IV) is the main bioactive component of Astragalus membranaceus (a traditional Chinese medicine) and has demonstrated significant potential in fields such as immune regulation, neuroprotection, anti-tumor effects, and organ protection. Studies have confirmed that AS-IV inhibits the viability and glycolysis of HCC cells by regulating KAT2A-mediated PGAM1 succinylation, highlighting its potential for combination therapy with existing HCC treatments (46).

## 6 Challenges and future perspectives

Succinylation, as a key post-translational modification, has emerged as an important regulator linking metabolic reprogramming and immune responses in cancer. It contributes to reshaping cellular metabolic networks and influencing immune cell functions through site-specific modifications, thereby helping to bridge metabolism and immunity in the tumor microenvironment. Recent advances highlight its potential in oncology, where dysregulation is associated with tumor progression and immune evasion.

In summary, lysine succinylation integrates metabolic regulation, immune modulation, and therapeutic intervention into a coherent framework by acting as a metabolite-driven molecular code that translates TCA-cycle flux and succinyl-CoA availability directly into functional changes in both tumor cells and immune compartments. Unifying principles emerge across the reviewed evidence: succinylation’s charge reversal and steric effects enable tighter coupling to the Warburg effect and TME nutrient stress than acetylation or phosphorylation. This allows bidirectional control by degrading PD-L1 to relieve checkpoint inhibition, while simultaneously promoting lactate accumulation and M2-like polarization to foster immunosuppression, thus establishing a self-reinforcing metabolism-immunity axis uniquely amenable to dual metabolic-immunotherapeutic targeting.

Nevertheless, current knowledge harbors important limitations. The regulatory architecture remains incomplete: writers and erasers are largely repurposed from other PTM or metabolic pathways, with only one histone-specific reader identified. Mechanistic studies rely predominantly on correlative succinylomics and cell-line models without establishing causality in heterogeneous tumor microenvironments. Furthermore, spatiotemporal dynamics driven by fluctuating succinyl-CoA levels and subcellular compartmentalization have not been systematically mapped at single-cell resolution, restricting generalizability beyond the specific cancer types investigated.

These gaps point to several key unanswered questions that should guide future research (1): whether additional non-histone readers exist and how they decode succinylation signals in diverse cellular contexts; in particular, the role of succinylation in less-explored immune cell populations, such as natural killer (NK) cells, B cells, and myeloid-derived suppressor cells (MDSCs), remains largely undefined. It is currently unclear whether succinylation modulates NK cell cytotoxicity, MDSC recruitment, or immunosuppressive activity, highlighting an important gap in our understanding of the tumor immune microenvironment (2); the extent to which succinylation modifications causally drive immunotherapy resistance or response in patient-derived models and clinical cohorts (3); the feasibility of developing succinylation-based biomarkers (e.g., site-specific PD-L1 or PKM2 succinylation levels) for patient stratification; and (4) optimal strategies for combining succinylation modulators (CPT1A activators, SIRT5 inhibitors, or succinate-loaded microparticles) with existing immune checkpoint blockade or metabolic drugs in rationally designed clinical trials (5); how to precisely target succinylation modulators while minimizing off-target effects on healthy immune cells. Given that succinylation is tightly coupled to central metabolic pathways, it remains unclear how therapeutic interventions can selectively modulate succinylation in tumor cells without impairing normal immune cell function. Future studies should address the development of context-specific targeting strategies and evaluate potential toxicity profiles in preclinical models (6); whether integrative multi-omics approaches, such as the combination of succinylomics with spatial transcriptomics or spatial proteomics, can enable high-resolution mapping of succinylation dynamics within the tumor immune microenvironment. Such approaches may provide critical insights into the spatial and cell-type-specific regulation of succinylation *in vivo* and (7) how artificial intelligence- or machine learning-based frameworks can be leveraged to integrate multi-omics datasets and predict succinylation-driven regulatory networks, although the reliability, interpretability, and biological validation of such models remain key challenges to be addressed. Addressing these questions through integrative multi-omics, spatial transcriptomics, and mechanism-focused preclinical validation will be essential to realize succinylation’s full potential as a bridge from metabolic reprogramming to precision cancer immunotherapy.
